# The Family Mental Health Cafés: A psychoeducation intervention to diminish stigma and isolation for families living with mental illness

**DOI:** 10.1192/j.eurpsy.2025.562

**Published:** 2025-08-26

**Authors:** C. C. Williams

**Affiliations:** 1 Factor-Inwentash Faculty of Social work; 2 Rehabilitation Sciences Institute, Temerty Faculty of Medicine, University of Toronto, Toronto, Canada

## Abstract

**Introduction:**

Psychoeducation is a well-supported intervention in psychiatry aimed at improving outcomes for patients with serious mental disorders and their families. It primarily focuses on enhancing family understanding of the illness, reducing stress, and fostering a supportive environment for the patient. However, traditional psychoeducation often emphasizes increasing caregivers’ capacity to manage the illness, rather than addressing the family as a unit coping with care needs and the stigma associated with mental illness. Family mental health cafés have been developed to address these broader issues.

**Objectives:**

The aim of this study was to explore the experiences of participants in family mental health cafés and evaluate its impact on feelings of stigma and isolation.

**Methods:**

The Family Mental Health Cafés were implemented in five Ontario cities from 2018 to 2019, these cafés were organized in collaboration with the Canadian Mental Health Association. They drew on the World Café and Death Café models, focusing on caregiving and care-receiving within the family unit and its interactions with the community. Discussions included managing illness and other stressors, successful strategies, and improvements needed for family well-being. Participants completed evaluations with both closed and open-ended questions

**Results:**

A total of 67 individuals participated, identifying as diagnosed individuals, family members, service providers, or combinations thereof. Sixty-six completed evaluations, with 99% finding the cafés well-planned and engaging, and 88% recommending them to others. Qualitative feedback emphasized the value of shared experiences, resource exchange, and diverse perspectives from patients, family members, and service providers. Participants appreciated the integration of these perspectives as a positive aspect of the café experience.

**Conclusions:**

The cafés offer a novel approach to psychoeducation by focusing on the well-being of the entire family, their mutual caregiving investments, and challenges in navigating social and institutional environments. Participants valued the process for addressing isolation, and engagement with others with similar experiences may have helped reduce stigma, though this was less clear. Future research could explore the long-term outcomes of single or repeated café experiences.

**Disclosure of Interest:**

None Declared

